# Spreading positive change: Societal benefits of meditation

**DOI:** 10.3389/fpsyt.2023.1038051

**Published:** 2023-04-12

**Authors:** Veronika Engert, Olga Maria Klimecki, Philipp Kanske, the Mindful Universities Research Group

**Affiliations:** ^1^Institute of Psychosocial Medicine, Psychotherapy and Psychooncology, Jena University Hospital, Friedrich Schiller University, Jena, Germany; ^2^Social Stress and Family Health Research Group, Max Planck Institute for Human Cognitive and Brain Sciences, Leipzig, Germany; ^3^Clinical Psychology and Behavioral Neuroscience, Faculty of Psychology, Technische Universität Dresden, Dresden, Germany

**Keywords:** attention, emotion, perspective-taking, meditation, social behavior, stress

## Abstract

Research over the past decades has revealed a variety of beneficial effects of meditation training. These beneficial effects span the levels of health and well-being, cognition, emotion, and social behavior. Around the same time, sociologists have shown that traits and outcomes on the individual level have the potential to spread in communities over three or more degrees. This means, for example, that changes can spread from one person to the next, and on to yet another person. Here, we propose that meditation-induced changes may likewise spread through the social networks of meditation practitioners. Such spreading may happen by positively influencing others through prosocial actions, improved cognitive functioning, and increased positive affect. Positive affective states and their underlying physiological correlates may also be shared in the literal sense. We argue that the spreading of positive meditation effects could provide the basis for collective responses to some of the urgent challenges we face in our current time and society and call for future meditation research to examine the phenomenon.

## Introduction

Modern societies have made great strides toward improving health, technology, and general knowledge. Next to these fundamental achievements, the limitations of modern societies have become overly apparent. We face dire economic and environmental crises, unprecedented psychosocial stress, and a plethora of associated diseases. Several of the negative side effects of today’s lifestyle, including loneliness and substance abuse, were amplified by the Covid-19 pandemic (1). Secularized meditation has emerged as one possible remedy to better cope with the challenges of our time (2). Aiming to build stress resilience and foster health and well-being, interventions such as the eight-weeks Mindfulness-Based Stress Reduction Program (3) and Mindfulness-Based Cognitive Therapy (4) have built a strong reputation in mainstream clinical and educational settings (2). The popularizing of the concept of mindfulness roots in Jon Kabat-Zinn’s initiative in the 1970s aiming to make Buddhist meditation accessible to secular audiences. As per Kabat-Zinn’s definition, mindfulness is “the awareness that arises through paying attention, on purpose, in the present moment, and non-judgmentally” (5). More recently, there is also growing interest in compassion-based interventions (6). Programs such as Compassion Focused Therapy (7) and the Mindful Self-Compassion Program (8) foster positive affect and promote social connection.

## Beyond the individualistic perspective –societal spreading of positive meditation effects

There is a steadily growing body of literature showing the positive effects of meditation practice on brain structure and function, cognition, affect, social behavior, and peripheral physiology of the active trainee [e.g., (9–13)]. However, long-term Buddhist practitioners and meditation teachers propose another level of change. They suggest that the positive influence of meditation can go beyond the individual level toward societal transformation, thus providing the basis for collective responses to the personal, societal, and ecological challenges we face (14, 15).

This may sound like a bold idea, but the spreading of phenomena through social networks is nothing new. Seminal research from observational and experimental datasets shows that numerous traits and outcomes covary within social networks, often spanning three degrees of separation between network members [i.e., from person to person to person; (16)]. A causal influence of one person’s attribute on the same attribute in others has been suggested for phenomena as diverse as obesity, smoking, alcohol and food consumption, sleep, drug use, happiness, loneliness, depression, divorce, and, in controlled laboratory experiments, cooperative behavior and political mobilization (16). Another vivid example for the spreading of phenomena stems from research in the workplace. Here, it was shown that ethical leadership, defined as the demonstration of normatively appropriate conduct, and the promotion of such conduct to followers, was related to extra effort and helping in followers especially at higher levels of follower moral emotions and mindfulness (17).

**Figure 1:**
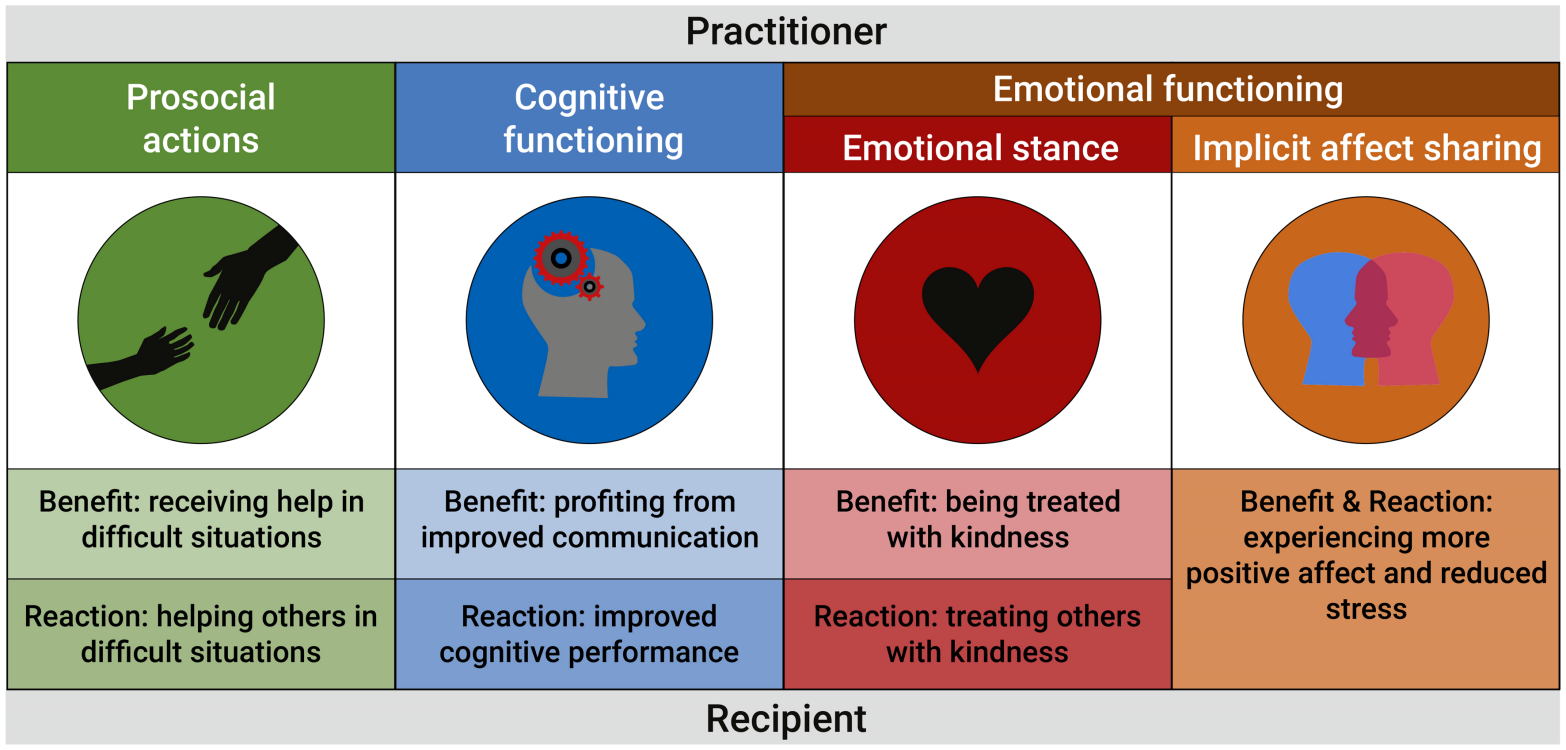
Schematic representation of pathways through which meditation effects may spread to others. As a basic mechanism, recipients may simply benefit from meditation-induced effects on the practitioner’s prosocial behavior, cognitive functioning and emotional functioning. They may additionally modify their own subsequent behavior, cognitions or emotions. In the most literal sense of the word spreading, the positive affective states and underlying physiological correlates of a practitioner may also be directly shared.

Our understanding of “spreading” in the current paper implies that there needs to be a causal process in which a meditation-induced effect in one person triggers the production of an effect in another person (also referred to as a recipient). However, it is not conditional that the two effects are isomorphic (i.e., similar in form). In the following sections, we summarize positive effects of meditation training on the practitioner, grouped by changes in a) prosocial behavior, (b) cognitive functioning, and (c) emotional functioning and stress sensitivity. In each section we then reflect on the different pathways through which these effects may spread to fellow human beings. As a basic mechanism, we suggest that recipients simply benefit from a meditation-induced effect. Responding to this benefit, they may additionally modify their own subsequent behavior, cognitions, or emotions. In the most literal sense of the word spreading, the positive affective states and underlying physiological correlates of a practitioner may also be isomorphically shared (see Figure 1 for a schematic representation). Our operationalization of spreading shares several features with what Kirby and colleagues describe as the “flow” of compassion (18). The authors emphasize that rather than being static, compassion has to be understood in a social-interactional context (19), in which it can “ripple.” In other words, if one is compassionate toward another, this may trigger the other to be compassionate toward themselves and others in turn (19–21). We conclude this paper with an argument for the future investigation of whether and how meditation effects spread in social networks.

## Prosocial actions toward others

### Summary of meditation effects

One pathway through which the effects of meditation may spread and improve the lives of others is prosocial behavior. If we practice to hone in on and nourish positive feelings toward others, we are bound to treat them with more respect and to deliberately reach out to those in need. The field has approached the topic of prosociality with a variety of different intervention programs and measures. For instance, after extensive training of up to nine months, Böckler and colleagues (22) showed that compassion-based practice increased altruistically motivated, prosocial behavior. Interestingly, much shorter compassion-or mindfulness-based interventions [e.g., (23–25)] also led to more prosocial behavior, even if meditation-naïve participants practiced only once for a few minutes (26, 27).

Despite the variability in how meditation training is administered, both face-to-face and purely online instructions seem to boost prosocial behavior (28, 29). Given that for many people attending a nine-months, in-person program is not realistically feasible, this finding is of great relevance when it comes to the scalability of interventions to large groups. Regarding interventions aimed at promoting the relations and interactions between groups, increasing evidence shows that both mindfulness- and compassion-based meditation can have a beneficial impact on intergroup relations [reviewed in (30)], proving helpful even in the reduction of outgroup prejudice among youths living amidst ethnic tension and violent conflict (31).

A crucial factor when assessing the effects of meditation interventions on prosociality is the question of how it is assessed. Studies on the prosocial effects of meditation-based interventions have utilized a large variety of measures, ranging from self-report to economic games to real-world behavior. There is some evidence that self-reports are more sensitive to interventions and show increases irrespective of the specific form of meditation that has been trained but do not necessarily correspond to changes in actual behavior as assessed with different types of economic games (22). An advantage of such economic games, that have been applied relatively widely, is that they allow the quantification of interaction behavior (25, 32). However, they have been criticized for being overly simplistic, and other more real-world assessments have been undertaken to complement them. For instance, after three weeks of an app-based mindfulness training, participants were more likely to help a stranger in pain (29). Further real-world prosocial improvements included reduced punishment of transgressors (33) and helping ostracized individuals (26, 27). Despite the diversity of assessment methods, there is relative accordance regarding the positive effects of meditation training on prosociality. Nevertheless, and as per a recent call by Mascaro and colleagues, there is a need for better integration of definitions and methodologies to make compassion research more “accurate, reliable, and transferrable,” and to promote synthesis of knowledge within and across fields (34).

Meta-analyses confirm the positive effects of meditation-based mental training on prosocial behavior (35, 36). At the same time, they reveal the need for future research to focus on the factors that moderate these effects. Three major groups of variables are being discussed (37). First, while secular mindfulness programs have contributed greatly to the success of the construct in the West, it has been suggested that incorporating ethics into a mindfulness intervention could strengthen its impact on prosociality (38). Despite some evidence for this notion (39), meta-analytic data synthesis clearly shows that effects on prosocial behavior evolve also without ethical training elements (40). Second, pre-training personality traits might influence training efficacy. This has been shown for intervention effects on well-being and distress (41, 42), but may also be the case for prosocial behavior (39). Third, relational aspects seem to be important. Embedding practitioners in social networks and incorporating these networks in the mindfulness-and compassion-based practice may enhance prosocial training effects (43).

### Potential pathways of spreading

Overall, different types of meditation training and ways of administering these trainings may boost different dimensions of prosociality, thus increasing the tendency of a practitioner to help others. This creates an active pathway of spreading through which the positive effects of meditation are voluntarily forwarded to a practitioner’s social groups and communities.

A possible reaction to receiving a prosocial act, which can lead to further spread, is to reciprocate it in the future [see theory of reciprocal altruism; (44)], thus creating a positive reinforcement loop of helping behavior. And not just the recipient but also witnesses or individuals indirectly learning about others’ acts of kindness may be encouraged to imitation. This assumption is confirmed by an extensive meta-analysis synthesizing several decades of research on prosocial modeling and showing that witnessing others’ acts of kindness inspires prosocial behavior through a process termed “prosocial goal contagion” (45). As is suggested in the context of ethical leadership in the workplace, especially individuals with higher levels of moral emotions and mindfulness are likely to engage in imitation or goal contagion (17). In these scenarios, changes in prosocial behavior in a practitioner would lead to an isomorphic state in the recipient (or observer). Alternatively, even if not motivated to demonstrate full-blown prosocial actions themselves, recipients or witnesses of others’ prosociality may react with positivity and happiness (46) –positive states that may, in turn, spread among their social contacts (see paragraph on emotional functioning).

## Cognitive functioning

### Summary of meditation effects

A second, and less deliberate pathway of spreading than active prosocial behavior may be through improved cognitive functioning. Indeed, meditation training has a positive impact on a range of cognitive abilities. A meta-analysis showed that mindfulness-based training particularly improves executive functions, including working memory, in older adults (47). The beneficial effect of meditation training on cognitive functions is corroborated by two other meta-analyses. The first showed that different types of meditation training improve executive control (48), while the other found that mindfulness-based interventions and long-term meditation practice are both associated with better attention performance (49).

There is some evidence for an impact of meditation interventions on perspective-taking, that is, the ability to infer or adopt the mental states of others (also known as “Theory of Mind”). This evidence is mostly based on self-report measures [for a systematic review in children and adolescents see (50)]. Few studies suggest that very brief interventions may already yield state changes in perspective taking (27, 51). Longer-term training in observing thoughts meditation and a contemplative dialog focusing on the ability of perspective-taking was shown to cause moderate effects on behavioral measures of perspective-taking, assessed in realistically complex tasks (52). In this study, mental improvement went along with increased cortical thickness in regions that are meta-analytically related to perspective-taking (53, 54), as well as with changes in the resting function of the brain (55). Taking the perspective of other people was also linked to metacognition (i.e., the capacity to think about and monitor one’s own cognitive processes (56)), which in turn was demonstrated to improve with meditation training (57). Eventually, these long-term changes in behavioral perspective-taking were related to the degree of change in participants’ self-perspectives (58). Thus, meditation seems to improve a general capacity to “mentalize” about others’ and one’s own states (59).

### Potential pathways of spreading

#### Attention

Improved attention may simply be transmitted to (or caught by) the interaction partners of practitioners. Along those lines, recent studies have discovered the phenomenon of attention contagion. It was shown that both in analog (60) and in digital learning situations (61), attentional states (attentive and inattentive in nature) spread among the members of a group. While distraction through inattentive others may be one mechanism contributing to the spreading of inattentiveness in particular (60), eye gaze, which is a proxy for visual attention (62), may be at the basis of attention spreading. Thus, research in the actual classroom environment demonstrates that teacher eye gaze toward students positively influenced student-rated agentic and communal learning (63).

#### Executive function

Executive function is a complex process involving a number of skills. It therefore seems rather unlikely that it would be contagious in the same sense as attention. Rather, we suggest that improvements in executive function change the way practitioners approach and interact with their fellow human beings on a daily basis. In the context of a teacher-student or parent-child relationship, for example, practitioners could become more flexible in thinking, and exhibit better self-control and organization. All of these attributes would likely boost successful teaching and parenting ability. Additionally, they may create an atmosphere that allows recipients to think more flexibly and controlled themselves, or even encourage imitation. A conference paper presented at the annual meeting of the German Economic Association provides indirect evidence for this notion (64). In an extensive survey across 22 countries, including approximately 200 teachers per country, the authors show that teachers’ literacy and numeracy skills, both of which are closely linked to executive function (65, 66), predict the respective skills in their students, independent of teacher salaries.

#### Perspective taking

On a similar note, improvements in perspective taking, that allow the practitioner to better perceive and understand the states and needs of others, may boost communication and flow of information between interaction partners, without necessarily triggering isomorphic states in the latter. Along those lines, a study by Gehlbach and colleagues (67) demonstrates how training-enhanced perspective taking abilities in teachers improved the teacher-student relationship as well as students’ classroom behavior and course competency scores. It has also been suggested that by increasing attentional capacities, interoceptive awareness, emotion regulation, and perspective taking all at the same time, mindfulness meditation changes the cognitive processing of morally relevant information, promoting moral action in consequence (68). Because it is an allocentric cognitive capacity allowing one to better understand the mental states of others and is directly related to prosocial behavior (69), the socio-cognitive capacity of perspective taking may be of particular importance when investigating the mechanisms by which meditation-induced cognitive benefits spread to others.

## Emotional functioning and stress experience

### Summary of meditation effects

Regarding stable traits assessed with self-report questionnaires, three months of compassion-based mental training yielded improvements in non-judgmental attitude, acceptance, and self-compassion (70). State ratings assessed in a socio-cognitive computer task showed increased behavioral compassion (52), also specifically after compassion-based training. More fine-grained state ratings assessed before and after daily meditation revealed prominent increases in positively valenced thoughts after compassion-based practice, and in positivity of affect and warmth after different types of practice, including mindfulness-, compassion- and thought-based techniques (71).

Chwyl and colleagues (72) highlight another approach to enhance self-compassion in particular. Negative beliefs about self-compassion (i.e., it leading to complacency or indulgency) are key barriers to people treating themselves compassionately. By targeting such negative mindset in concrete online messages, the authors could predict an increase in self-compassionate responding 5 to 7 days later. Thus, while this approach does not rely on mental training, it offers great opportunity to reach many people with minimal expenditure.

While stress involves more than one emotion, it refers to experiences that cause feelings of anxiety and frustration by pushing us beyond our ability to successfully cope (73). Along with an emotional component, the typical stress response entails activation of the sympathetic nervous system and the hypothalamic–pituitary–adrenal axis, resulting in cortisol release (74). Meditation was shown to buffer the negative emotional aspects of psychosocial stress, irrespective of practice type (reviewed in 75). Cortisol reactivity to acute psychosocial stress was selectively reduced through the cultivation of compassion- and thought-based techniques (76).

### Potential pathways of spreading

As is the case for the emotional state of happiness, which was shown to extend up to three degrees within social networks, and not merely as a result of individuals associating with similar individuals (77), a meditation-induced positive and stress-free stance toward oneself, others, and toward life in general may spread among the social contacts of practitioners. Actual sharing is a possible mechanism through which such spreading of positivity and reduced stress may occur. Research over the past two decades has impressively shown that, while humans may perceive themselves as autonomous entities, they often share the affective, neural, and peripheral-physiological states of their fellow human beings (78, 79). The occurrence of such interindividual sharing seems to depend on levels of empathy, that is, on the extent to which the perception of another’s emotional state leads to a similar emotional state in the perceiver (80, 81). Most of the work examining empathy-dependent sharing has focused on instances of negative experience. Prominent examples are studies on the neural correlates of empathy for disgust and pain. In detail, Wicker and colleagues (82) found that observing the facial expression of disgust in others and feeling disgusted oneself activated the same sites in the anterior insula and anterior cingulate cortex. In a similar manner, observing a loved one receive a painful stimulus and experiencing pain oneself activated nearly identical sites in the anterior insula, rostral anterior cingulate cortex, brainstem, and cerebellum. Anterior insula and anterior cingulate cortex activation for others’ pain were also correlated with observers’ empathy scores (83, 84).

The topic of physiological sharing was taken up in the context of stress research. Here it was found that the mere observation of a target undergoing psychosocial stress resonates to the level of a physiological stress response in the observer [for a review see (78)]. The extent to which an observer’s physiological activation was proportional to that of the stressed target was dependent on the emotional and spatial closeness of the observer-target dyad, and individuals with higher self-reported empathy again showed higher stress resonance (85).

However, empathic sharing is not limited to instances of negative experience. Both the first-hand experience and the second-hand observation of pleasant touch have been found to trigger overlapping avtivity in the medial orbitofrontal cortex (86). Considering this literature, we hypothesize that the positive affective and stress-reducing effects of meditation, including their underlying biological states, are likewise shared between individuals –a mechanism of spreading that implies the recipient to show isomorphic changes in his or her own affective state.

We additionally hypothesize that the positive emotional transformations may spread and benefit others by making practitioners less absorbed by their own commitments and daily hassles, more understanding of others’ shortcomings, and thus more capable and willing to show emotional support. Work on moral expansiveness showed that the scope of entities deemed worthy of moral concern was reliably linked to individuals’ compassion levels (87, 88). In light of these findings, it could even be possible that training compassion would cause a widening of the practitioner’s moral circle. On a more profound level of spreading, a recipient may become motivated to imitate emotional positivity, a compassionate stance, and a stress-free attitude.

## The immediate effects of meditation are not always positive

In recent years, and partially owed to an overly simplistic presentation of what mindfulness can achieve in the popular media, an undifferentiated view of mindfulness as the panacea for all the ills of modern life has developed. We want to emphasize that, while meditation has an immense potential for positive change, it is by no means a cure for everything. Meditation can for example, help with stress reduction, but without further action, meditation alone cannot change the problems that are at the root of our overly stressful lifestyle. Also, contrary to the general belief that meditation is always relaxing, it requires effort and may increase physiological arousal (89). Meditation training can also trigger adverse effects. A study carried out in a population-based sample of over 400 participants in the United States reported that 30–50% of respondents indicated the occurrence of meditation-related adverse effects (90). Among those, anxiety, traumatic re-experiencing, and emotional sensitivity were among the most common (90). Another cross-sectional study with over 1,000 participants found that 26% of meditators reported particularly unpleasant experiences that may have been caused by meditation (91). These unpleasant experiences may seem counter-intuitive at first glance. But meditation training relies on refining the perception and acceptance of thoughts, emotions, and sensations. If previously suppressed, the occurrence of negative experiences should not come as a surprise. Therefore, just as there needs to be sensitivity about bodily ailments when starting a physical training regimen, awareness of the possibility of unwanted side effects and trauma-sensitivity are warranted to minimize the possible risks of meditation (90). Importantly, it seems that especially deconstructive types of meditation (e.g., vipassana/insight meditation) rather than attentional (e.g., mindfulness of breathing) or constructive types (e.g., loving-kindness meditation) are prone to trigger unwanted side effects. Thus, preparing for the challenging experiences that may arise from deconstructive practice by initially focusing on attentional and constructive exercises, and cultivating meta-awareness and acceptance, may shield new meditators from such unwanted side-effects (59).

## Factors influencing the spreading of meditation effects

We do not wish to suggest that spreading would be a ubiquitous response that arises at all times and under all circumstances. Rather, numerous factors related to the individual, the practice, and the environment are likely to influence whether or to which extent spreading occurs. Personality traits are one example. On the side of the practitioner, they influence training efficacy, and thus the intensity of a “transmission signal.” On that note, an influence of pre-practice personality was shown for intervention effects on well-being and distress ((41), (42)), but may also exist for prosocial behavior (39). Also, levels of self-confidence may modulate the degree to which practitioners exert an influence on their interaction partners, as was shown regarding the influence of individual A’s confidence in the likelihood of a reward on individual B’s expectation of that reward (92). Somewhat relatedly, derived from research on the position of leaders as role models for what is considered appropriate behavior (93), one would expect a stronger transmission signal from individuals in a position of power and leadership. Likewise, recipient personality is bound to play a role. Previous studies accordingly demonstrate the role of moral emotions and mindfulness in the extent to which ethical leadership is related to extra effort and helping in followers (17), and to which empathy modulates pain and stress contagion (83, 84, 94).

Practice type might be another influencing factor. While we suggest that the effects of various types of meditation have the potential to spread, practices from the constructive family may be particularly powerful. Specifically, as categorized by Dahl and colleagues (59), relationship-oriented practices that cultivate healthy and constructive relationships as well as value-oriented practices that promote incorporating ethical principles into ones views may promote spreading due to their direct focus on others rather than the self. With respect to environmental aspects, contact frequency among the members of a social network is one obvious example for a factor influencing the likelihood of spreading. Thus, emotions were shown to spread much like infectious diseases, with the number of contacts determining the probability of a specific emotion to spread or not (i.e., the probability of becoming content was shown to increase by 0.02 per year for each contact that was content, and the probability of becoming discontent by 0.04 per year per discontent contact) (95).

## Summary and outlook

There are multiple ways through which meditation-induced positive change may spread from practitioners to their social networks. Meditation effects in the practitioner, whether behavioral, cognitive, or emotional, may be transmitted through their immediate positive benefit on the environment. Responding to this benefit, recipients may also modify their own behavior, cognitions, or emotions. Positive affective states and their underlying physiological correlates may also be literally shared (Figure 1). In all scenarios, important values such as kindness, compassion, and resilience may be passed on.

In the instances of spreading depicted here, we focus on the spreading of positive meditation effects, not on the spreading of meditation itself. Alternatively, if practitioners deliberately passed on their contemplative knowledge (e.g., an academic teacher offering a mindful breathing exercise in a lecture break), then the actual meditation experience, along with its direct effects (e.g., improved attention), would be passed on. Practitioners could also openly express their positive experiences with meditation, or else, others could consciously perceive that positive changes in practitioners are due to their meditation practice. In these instances, it would likely be the motivation to meditate that would spread between individuals, and consequently multiply beneficial meditation effects.

A better understanding of the means by which the effects of meditation propagate through social networks may reveal opportunities to increase this transmission. Thus, testing whether and how the effects of meditation training spread in our society could be a fruitful next step in meditation research. Based on the insight that the traits and strengths of individual teachers explain a great proportion of the variance in their students’ achievements (96), we argue that the education system, ranging from nurseries to schools and universities, offers a particularly relevant venue in which to take those steps. Ultimately, to maximize the societal benefits of meditation, we should aim to investigate the spreading of meditation effects through other systems, including companies, hospitals, and conflict zones. We suggest that this novel approach would motivate new ways of addressing urgent topics of our time and society. Strategically teaching meditation in key positions across educational, health, economic, and political domains may infuse society with the necessary values and willingness to foster positive transformation.

## Author contributions

VE, OMK, and PK developed the theoretical framework and drafted a first version of the manuscript. All authors of the Mindful Universities Research Group critically revised the manuscript.

## Conflict of interest

The authors declare that the research was conducted in the absence of any commercial or financial relationships that could be construed as a potential conflict of interest.

The reviewer A-LS declared a shared affiliation with the author VE to the handling editor at the time of review.

## Publisher’s note

All claims expressed in this article are solely those of the authors and do not necessarily represent those of their affiliated organizations, or those of the publisher, the editors and the reviewers. Any product that may be evaluated in this article, or claim that may be made by its manufacturer, is not guaranteed or endorsed by the publisher.
