# Supplementary material for: Spreading positive change: Societal benefits of meditation
Source: Front Psychiatry. 2023 Apr 12;14:1038051. doi: 10.3389/fpsyt.2023.1038051 (PMC10130585; doi:10.3389/fpsyt.2023.1038051)
Supplement: Supplementary file 1 [file Table_1.docx]

**Mindful Universities Research Group**

| Albrecht, Reyk | Department of Ethics in Sciences, Friedrich Schiller University and Institute of History, Theory and Ethics of Medicine, Jena University Hospital, Germany |
| --- | --- |
| Dobel, Christian | Department of Otorhinolaryngology, Institute of Phoniatry and Pedaudiology, Jena University Hospital, Germany. |
| Döring, Nicola | Institute of Media and Communication Science, Ilmenau University of Technology, Ilmenau, Germany |
| Engert, Veronika | Institute of Psychosocial Medicine, Psychotherapy and Psychooncology, Jena University Hospital, Friedrich-Schiller University, Jena, Germany |
| Guntinas Lichius, Orlando | Department of Otorhinolaryngology, Institute of Phoniatry and Pedaudiology, Jena University Hospital, Germany. |
| Haueisen, Jens | Institute of Biomedical Engineering and Informatics, Faculty of Computer Science and Automation, Technische Universität Ilmenau, Ilmenau, Germany |
| Kanske, Philipp | Clinical Psychology and Behavioral Neuroscience, Faculty of Psychology, Technische Universität Dresden, Dresden, Germany |
| Sandbothe, Mike | Department of Social Work, University of Applied Sciences Jena, Jena, Germany |
